# Isoform expression patterns of EPHA10 protein mediate breast cancer progression by regulating the E-Cadherin and β-catenin complex

**DOI:** 10.18632/oncotarget.15910

**Published:** 2017-03-06

**Authors:** Ye Li, Lu Jin, Fei Ye, Quanfu Ma, Zongyuan Yang, Dan Liu, Jie Yang, Ding Ma, Qinglei Gao

**Affiliations:** ^1^ Cancer Biology Research Center, Tongji Hospital, Tongji Medical College, Huazhong University of Science and Technology, Wuhan, Hubei, China; ^2^ Department of Oncology, Lombardi Comprehensive Cancer Center, Georgetown University, Washington DC, USA; ^3^ Department of Neurosurgery, Tongji Hospital, Tongji Medical College, Huazhong University of Science and Technology, Wuhan, Hubei, China

**Keywords:** EphA10s, EphA10, E-Cadherin complex, breast cancer, lymph node metastasis

## Abstract

Overexpression of EPHA10 protein was reported in concomitance with clinical severity of breast cancer. In this study, we annotate overexpression of EPHA10 protein with changes of isoform expression as EphA10s (EPHA10 isoform 2) and EphA10 (EPHA10 isoform 3). In the process of malignant transformation, secretory protein EphA10s is in low expression, and pseudo-kinase EphA10 is overexpressed and cytoplasmically enriched. Down-regulated EphA10s blunts stabilization of membrane-associate β-catenin via the interaction with ephrin A5. Cytoplasmic EphA10 maintains phosphorylation of E-cadherin. Restoring isoform expression pattern by up-regulated EphA10s and down-regulated cytoplasmic EphA10 inhibits cell invasion and lymph node metastasis by strengthening the stability of the complex of E-cadherin and β-catenin in membrane. Taken together, we defined the novel interaction via expression patterns of EphA10s and EphA10 that promote malignant transformation of breast cancer, and demonstrated the potential benefit in clinical usage.

## INTRODUCTION

Breast cancer (BC) is the most frequently diagnosed cancer among women (American Cancer Society) [1]. Heterogeneity of BC has been documented in BC diagnoses and therapies over the past two decades. The identification of heterogeneous biomarkers and molecular-driven therapies benefits patients with individualized treatment [2–3]. Though outcomes of BC have been improved due to this understanding, challenges still remain because of the aggressive nature, lack of representative biomarkers and potentially curable targets [4–6].

Inter-independent genetic profiles reported that the gene amplification of EPHA10 is between 0.8%-17.2% in breast cancer, and a missense mutation in the RTK domain (TCGA data) in 0.2% (5/482) of BC cases [7–9]. However, the EPHA10 protein is observed to be overexpressed in all BC subtypes regardless of their hormone status [7, 10]. The function and mechanism of the EPHA10 protein remains unclear. EPHA10 belongs to the Eph receptor tyrosine kinase family which characters with dual-signaling transduction of forward signaling (tyrosine kinase activity via Eph receptor) and reverse signaling (receptor-ligand interaction via ephrin ligand) [11]. Importantly, several lines of researches demonstrate that the transduction approach is implicated in the cell-cell interaction, tissue development, and tumor progression [12, 13]. Due to an altered β7-asparagine in the receptor tyrosine kinase (RTK) domain, the RTK of EPHA10 protein is a pseudo kinase, and thus, phosphorylation of substrate is blunted [14–16]. Overexpression of EPHA10 protein can be annotated by two isoforms (Supplementary Figure 1). EphA10s (NP_775912.2) is a small protein with a single Eph receptor A10 domain, which is predicted as a secretary protein (UniProtKB-Q5JZY3). EphA10 (NP_001092909.1) is a type I membrane protein with Eph receptor A10 domain, Fibronectin type III domain, and the pseudo-RTK domain. In this study, we investigated the expression pattern of EPHA10 isoforms and their biological functions in BC development and progression.

In the malignant transformation of breast cancer, E-cadherin (ECAD) and β-catenin are classically regarded as key players in cell-cell adhesion, and regulator in dynamic behavior [17, 18]. Notably, their expression and distribution were identified as biomarkers of breast cancer stem cells [18, 19–20]. ECAD plays a role in mediating calcium-dependent cell-cell adhesion, where the intercellular domain of ECAD binds to components such as β-catenin and p120-catenin, and associates with reorganization of actin cytoskeleton [21]. Under certain conditions, ECAD is phosphorylated (pECAD) which activates ECAD endocytic pathways and results in disassociation of ECAD complex [22, 23]. Loss of ECAD expression in membrane is one of the characteristic events of epithelial mesenchymal transition [24]. β-catenin has dual roles in the regulation of cancer progression. On one hand, β-catenin binds to ECAD in membrane where it facilitates cell-cell adhesion [25]. On the other hand, isolated from the ECAD complex (non-canonical pathway) [26] or from the Wnt signaling pathway (canonical pathway) [27], unbounded β-catenin acts as a strong oncogenic transcription factor [28]. In this study, we demonstrated that the expression pattern from EphA10s and EphA10 in BC weakens the stability of the membrane complex of ECAD and β-catenin, thus, promotes dynamic behavior of cancer.

## RESULTS

### Expression of EPHA10 protein in breast cancer

**Figure 1:**
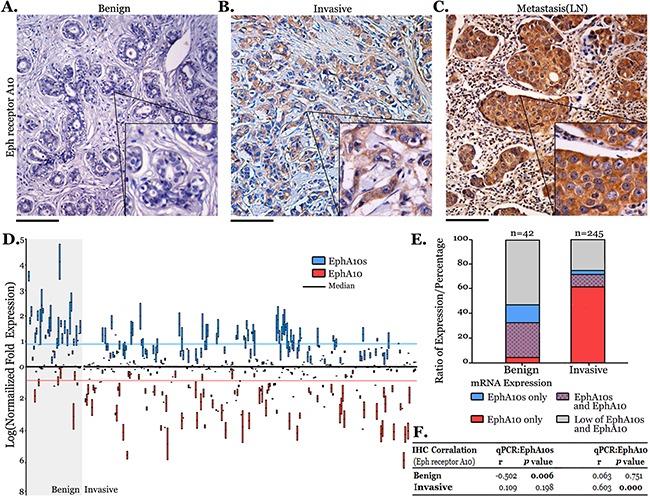
EPHA 10 expression in breast cancer Eph receptor A10 staining (IHC) was performed and representative images are shown as **(A)** benign sample, **(B)** invasive sample and **(C)** lymph-node sample with metastasis. The inset is 2×; scale bar: 100 μm. **(D)** Expression of EphA10s and EphA10 in 42 benign samples and 245 invasive samples. Statistic: Kruskal-Wallis, Median±Maxi/Mini are shown. **(E)** The proportion of EphA10s and EphA10 expression in clinical samples. **(F)** Spearman's correlation coefficients of Eph receptor A10 staining with EphA10s and EphA10 expression in clinical samples.

Expression of EPHA10 protein was screened by IHC staining with an anti-Eph receptor A10 domain antibody (Figure 1A-1C). In invasive samples (n=325), EPHA10 protein is highly expressed in cytoplasm in compared to benign samples (n=76), and in lymph-node metastasis samples (n=50), a significantly additional expression of EPHA10 protein was observed (Supplementary Table 1). Moreover, our data supported that EPHA10 protein is over expressed in samples with TNM stage severity (Supplementary Table 2).

Measuring EPHA10 protein expression by immunostain could be oversimplified because it is difficult to differentiate isoforms as EphA10s and EphA10. Therefore, we performed qPCR to identify mRNA expression of EphA10s and EphA10 in qualified 42 benign samples and 245 invasive samples. EphA10s is down regulated (*P*<0.001) while EphA10 is highly expressed (*P*<0.001) in invasive samples, and expression pattern of EPHA10 isoforms shifts from EphA10s/EphA10 (42.9%/33.3%) in 42 benign samples to EphA10s (13.6%) with dominantly expression of EphA10 (70.6%) in invasive samples (Figure 1D-1E, and Supplementary Table 3). Correlating IHC staining with expression of EphA10s and EphA10, we found a closer relation with EphA10s (r=-0.502, *P*=0.006) in benign samples, and with EphA10 (r=0.603, *P*<0.001) in invasive samples (Figure 1F).

### EphA10s and EphA10 expression patterns associate with breast cancer outcomes

In invasive samples, EphA10s is low expressed regardless of age, tumor size, lymph-node metastases, TNM stages, or cancer subtypes (Table 1). Differently, EphA10 is generally up-regulated and significantly increased in tumor-size enlargement (*P*=0.042) and TNM stage severity (*P*=0.002). Moreover, the expression level of EphA10s and EphA10 was found to contribute to the outcome in 5-year follow-up (Supplementary Figure 2). Patients with higher Eph receptor A10 staining show a poor expectation in disease-free survival analysis and in overall survival (OS) analysis (*P*=0.015, Log Rank HR=1.760 [95% CI: 1.071- 2.893]). When detailed by EphA10s and EphA10, higher EphA10s expression associates with a better disease-free and OS period, while higher EphA10 expression suggests a poor disease-free survival period and a poor OS expectation (*P*=0.013, Log Rank, HR=1.975 [95% CI: 1.157 -3.371]).

**Table 1:** Profile of EphA10s and EphA10 expression in breast cancer characters

Traits		qPCR:EphA10s		*P* value		qPCR:EphA10		*P* value
		High	Low			High	Low	
	n	n(%)	n(%)		n	n(%)	n(%)	
**AGE**	245				245			
≥45	178	24(13.5)	154(86.5)	0.667	178	128(71.9)	50(28.1)	0.053
<45	67	7(10.4)	60(89.6)		67	45(67.2)	22(32.8)	
**pT**	245				245			
T1 and T2	203	26(12.8)	177(87.2)	1.000	203	149(73.4)	54(26.6)	**0.042**
T3 and T4	42	5(11.9)	37(88.1)		42	24(57.1)	18(42.9)	
**pN**	245				245			
N0	203	29(14.3)	174(85.7)	0.125	203	138(68.0)	65(32.0)	0.062
N1 and N2	42	2(4.8)	40(95.2)		42	35(83.3)	7(16.7)	
**pSTAGE**	245				245			
I and II	210	23(11.0)	187(89.0)	0.058	210	141(67.1)	69(32.9)	**0.002**
III	35	8(22.9)	27(77.1)		35	32(91.4)	3(8.60)	
**Molecular Typing**	236				236			
Luminal A and B	134	12(9.00)	122(91.0)	0.159	134	98(73.1)	36(26.9)	0.207
HER-2	80	14(17.5)	66(82.5)		80	54(67.5)	26(32.5)	
Basal-like	22	2(9.1)	20(90.9)		22	19(86.4)	3(13.6)	

Tumor-node-metastasis (TNM) is classified according to the revised American Joint Committee on Cancer TNM classification. *P* value is evaluated with Pearson Chi-square, two tails.

### Secretary EphA10s inhibits cell migration and invasion

**Figure 2:**
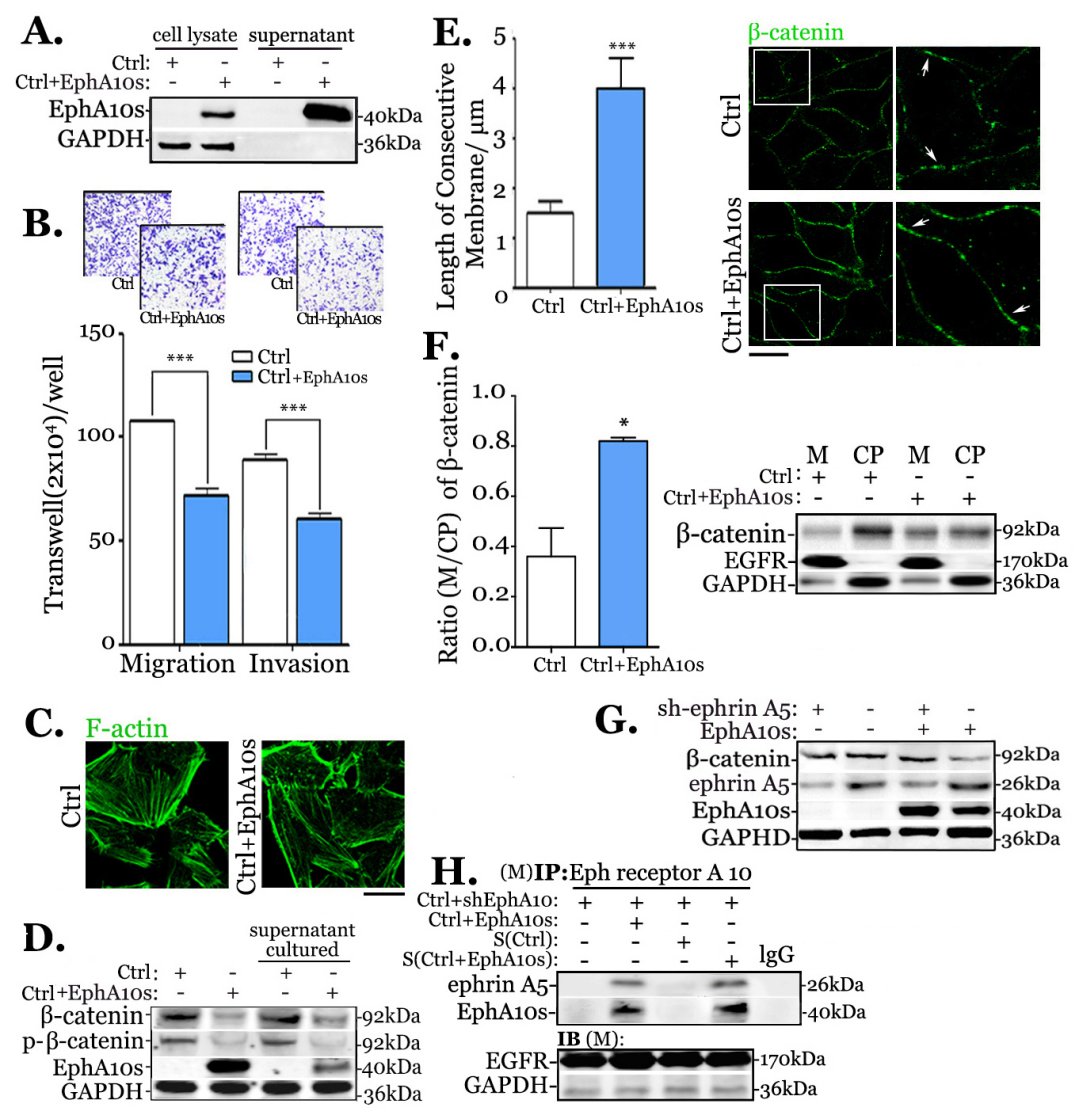
Secretary EphA10s inhibits migration and invasion by stabilizing of membrane-associate β-catenin in MDA-MB-231 cells **(A)** EphA10s was determined in cell lysates and supernatants from control (Ctrl, low EphA10 expression) cells and cells with lentiviral overexpression EphA10s (EphA10s) in Western Blots. **(B)** Measurement of migration and invasion was performed in Ctrl cells and cells with EphA10s expression (blue) in transwell chamber for 24 hours, arbitrary images from 3 repeats are shown. **(C)** Representative immunofluorescent images of F-actin of Ctrl cells and EphA10s expressing cells. **(D)** The total expression level and phospho-version of β-catenin were detected by Western Blot in cells with EphA10s expression or treated with EphA10s-containing supernatant. **(E)** Measurement of immunostain β-catenin in membrane in Ctrl cells and cells with EphA10s expression, representative image is shown, insert is 5×; Scale bar: 20 μm. **(F)** Measurement of β-catenin ratio of membrane-associate (M) over cytoplasmic (CP), which is analyzed based on 3-independent-immunoblot repeats. **(G)** Ctrl cells and EphA10s expressing cells with/without ephrin A5 silencing (sh-ephrin A5) were subjected to β-catenin analysis in Western Blot. **(H)** In EphA10 down-regulated MDA-MB-231 cells, anti-Eph receptor A10 IP was performed to analysis ephrin A5 and EphA10s in Ctrl cells and cells with EphA10s expression, and cells cultured with medium from Ctrl and EphA10s expressing cells. GAPDH and EGFR were used as a loading control. * *P*<0.05, *** *P*<0.001, statistic: two-tailed Student's t-tests. Mean±SD is shown.

EphA10s was down regulated in MDA-MB-231 cells (invasive) in compared to MCF-10A cells (benign, Supplementary Figure 3). To verify EphA10s as secretary protein, cell lysate (CL) and supernatants (S) of MCF-10A cells, and MDA-MB-231 cells with lentiviral EphA10s overexpression, were subjected to Western Blot (Figure 2A and Supplementary Figure 4A). Both natural EphA10s (MCF-10A) and overexpressed EphA10s are detected in culture supernatants. Moreover, EphA10s expressing MDA-MB-231 cells displayed a concomitant decrease in migration by 0.46 fold and in invasion by 0.33 fold (Figure 2B). When culture MDA-MB-231 cells with supernatant from EphA10s-expressing cells, the similar dynamic decrease was observed (Supplementary Figure 4B).

### EphA10s assists with β-catenin distribution and stability in membrane

Overexpression of EphA10s is found in resembling of F-actin filaments (Figure 2C). Molecular analysis in regulation of actin cytoskeleton was performed accordingly. EphA10s overexpression results in decrease of total β-catenin expression and the level of its phosphorylated version, while the similar findings were observed in cells cultured with supernatant from EphA10s-expressing cells (Figure 2D). Total β-catenin is explained by its subcellular accumulation. Immunocytochemistry staining shows that accumulation of β-catenin enhances in membrane in the present of EphA10s. The measurement of β-catenin in consecutive membrane staining shows a significant increase by 2.3 fold (*P*<0.001, Figure 2E). Furthermore, we enriched protein from membrane (M) and cytoplasm (CP), respectively. Immunoblot shows that accumulation of β-catenin increases in membrane and apparently decreases in cytoplasm in the presence of EphA10s, in which the β-catenin ratio of membrane-associate over cytoplasmic is enhanced by 2.7 fold (*P*=0.034, Figure 2F), while the total β-catenin shrinks.

### Ligand ephrin A5 participates in the redistribution of β-catenin

The interaction of EphA10s needs to bind with its ligands [11]. Proteins from ephrin family were analyzed in affinity and kinetic activity with Eph receptor A10 [14, 29]. ephrin A3, ephrin A4 and ephrin A5 shows the best performance based on the evaluation in structural and chemical simulation. We, thus, screened expression of ephrins in benign and invasive samples. ephrin A5 presents a significantly decrease in cancer, in which it shows an intensive correlation with the co-expression of EphA10s (r=0.558, *P*=0.004; Supplementary Figure 4C and 4D). Therefore, we knocked down the expression of ephrin A5 in cells. Immunoblot shows that the inhibition of total β-catenin in present of EphA10s is rescued in low-ephrin A5 expressing cells (Figure 2G). To verify the interaction, membrane protein from EphA10s^−^/ EphA10^−^ cells were subjected to anti-Eph receptor A10 immunoprecipitation (IP, Figure 2H). The blots of ephrin A5 shows in cells both with endogenous EphA10s or extraneous EphA10s.

### Overexpression of EphA10 accumulates in cytoplasm and promotes invasion

**Figure 3:**
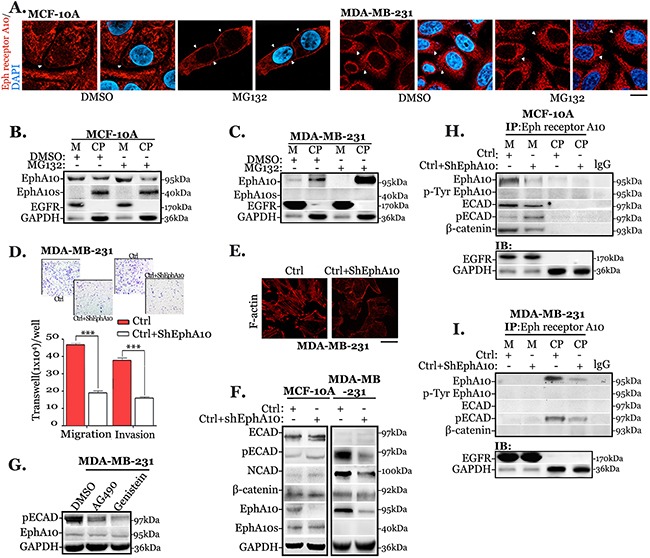
Cytoplasmic EphA10 promotes invasion and migration by associating with ECAD phosphorylation **(A)** Immunofluorescent staining of Eph receptor A10 in MG132 treated MCF-10A cells and MDA-MB 231 cells (4 hours), in which membrane staining is indicated by white arrows, scale bar: 20 μm. In the same scenario, **(B)** MCF-10A cells and **(C)** MDA-MB 231 cells were subjected to EphA10 expression in membrane (M) and cytoplasm (CP) in Western Blot. **(D)** Measurement of migration and invasion was performed in cells with Ctrl cells (red) and EphA10 knocking down cells (Ctrl+sh-EphA10) in transwell chamber for 24 hours, arbitrary images from 3 repeats are shown. **(E)** Representative immunofluorescent images of F-actin in Ctrl cells and EphA10 knocking down cells. **(F)** MCF-10A cells and MDA-MB-231 cells were subjected to the analysis of ECAD, pECAD, NCAD, and β-catenin when knocking down EphA10 expressing in Western Blot. **(G)** Immunoblot of pECAD was evaluated in MDA-MB-231 cells after treated with AG490 (20μM) and Genistein (50μM) for 6 hours. Anti-Eph receptor A10 IP was performed in MCF-10A cells **(H)** and MDA-MB-231 cells **(I)** to analysis the interaction with ECAD, pECAD and EphA10, p-EphA10 in membrane (M) and cytoplasm (CP). GAPDH and EGFR were used as a loading control. *** *P*<0.001, statistic: two-tailed Student's t-tests. Mean±SD is shown.

Using anti-Eph receptor A10 antibody, IHC staining is strongly accumulated in cytoplasm of invasive and metastatic samples, where the staining is closely correlated with EphA10 expression. We investigated the distribution *in vitro* (Figure 3A). In MCF-10A cells, EphA10 is accumulated in the membrane after treated with proteasome inhibitor MG132. Differently, in MDA-MB-231 cells, it shows an apparently cytoplasmic distribution, which is even stronger after using MG132. The observation was verified in Western Blot (Figure 3B and 3C). Knocking down EphA10 expression, the capability of migration and invasion in invasive cells is significantly decreased by 1.6 fold (migration) and 1.3 fold (invasion), with a concomitant depolymerization and disruption of F-actin (Figure 3E). However, down-regulation of EphA10 in MCF-10A cells results in less change of similar dynamic behaviors (data not shown).

### EphA10 associates with ECAD phosphorylation

For a better understanding the difference, both benign cells and invasive cells were subjected to further investigation. In Western Bolt (Figure 3F), knocking down EphA10 by lentiviral infection in MCF-10A cells leads to a slightly increase of pECAD, while in MDA-MB-231 cells results in a shrink of pECAD with a decrease of N-Cadherin (NCAD). The phosphorylation of ECAD complex involves the tyrosine kinase induced tyrosine phosphorylation [23]. We blocked the activity of tyrosine kinase by using inhibitors AG490 and Genistein [30] for 6 hours (Figure 3G). Immunoblot shows that both inhibitors notably blunt pECAD level without decreasing EphA10 expression.

EphA10 is redistributed to cytoplasm in invasive cells. We performed anti-Eph receptor A10 IP to understand the EphA10 interaction in either membrane or cytoplasm in both benign and invasive cells (Figure 3H and 3I). In both cellular fractionations, pseudo-RTK EphA10 shows a low activity of phosphorylation. In MCF-10A cells, membrane-associate EphA10 recruits the complex of ECAD and β-catenin, while low interaction is detected in cytoplasm. Knockdown EphA10 from membrane abolishes the interaction, in which ECAD associated with enhanced level of phosphorylation. In MDA-MB-231 cells, cytoplasmic EphA10 associates with pECAD, EphA10 knocking down weakens the interaction.

### Up-regulating EphA10s and down-regulating EphA10 inhibits cell invasion

**Figure 4:**
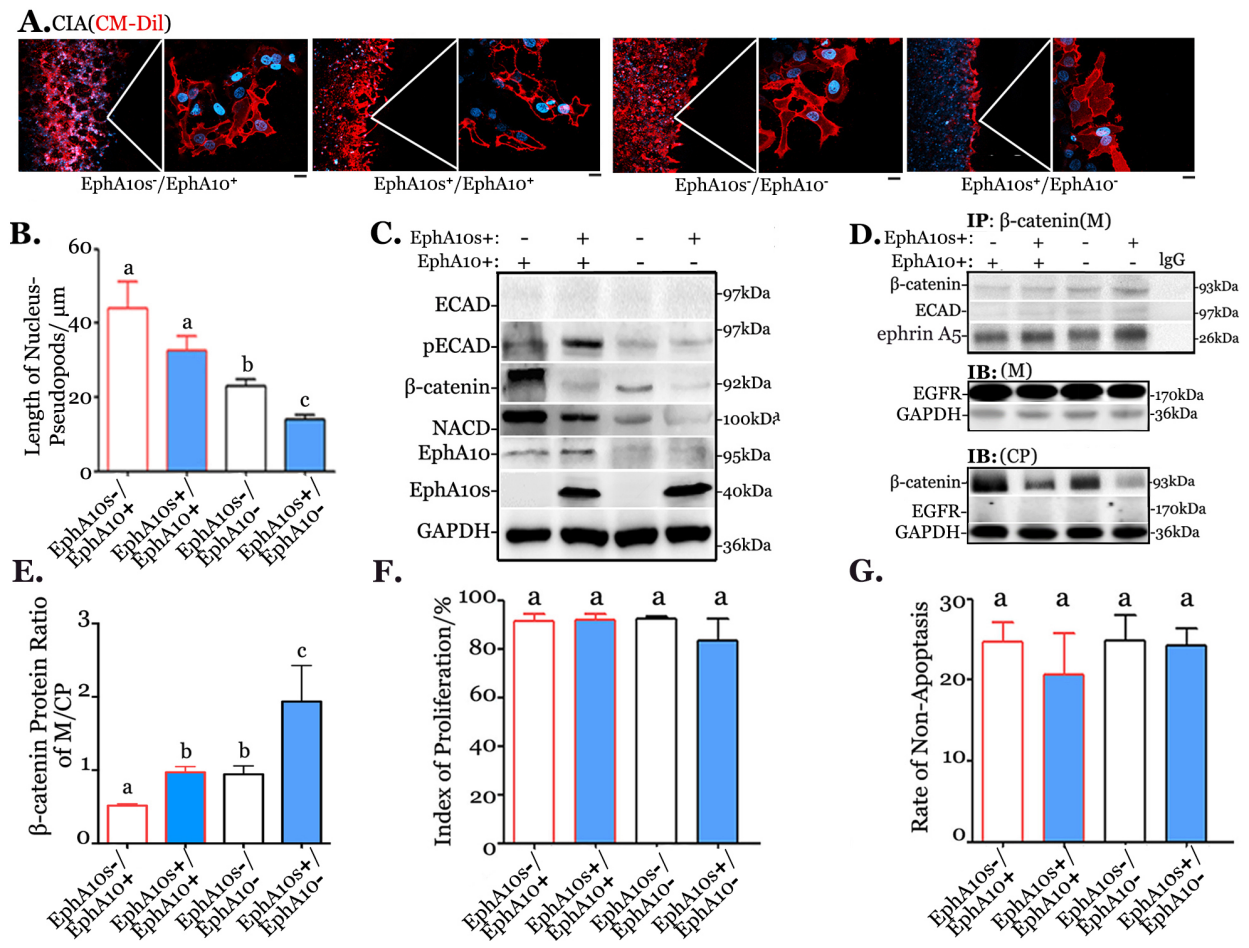
Expression pattern EphA10s and EphA10 regulates cellular migration and invasion Expression of EPHA10 protein was modified in MDA-MB-231 cells as high or low EphA10s (EphA10s+/−) with high or low EphA10 (EphA10+/−). **(A)** CIA was performed in cells with different EphA10s and EphA10 expression patterns. Arbitrary field from 3 repeats is shown, insets are 5×, scale bar: 20 μm. **(B)** In the same scenario, measurement of the length from a nucleus to its pseudopods was analyzed in cells. Cells were subjected to analysis of ECAD, pECAD, NCAD, and β-catenin in Western Blot **(C)**, to analysis of interaction with β-catenin, ECAD and ephrin A5 in membrane (M) and in cytoplasm (CP) with anti-β-catenin IP **(D)**. GAPDH and EGFR were used as a loading control. **(E)** Measurement of β-catenin ratio in membrane-associate over cytoplasmic. **(F)** Measurement of cells proliferation by using CFSE stain, and **(G)** measurement of annexin V/ propidium iodide based apoptosis assay were performed in cells. Mean±SD is shown.

We modified expression patterns of EphA10s and EphA10 in MDA-MB-231 cells by lentiviral infection for a further investigation. Expression patterns were identified as high/low EphA10s (EphA10s^+^/EphA10s^−^) and high/low EphA10 (EphA10^+^/EphA10^−^), respectively. Compared to EphA10s^−^/EphA10^+^ cells, silencing EphA10 results to 0.62-fold decrease in the ability of invasion, with additional expression of EphA10s, the invasive ability collapses to 0.33 fold (ANOVA, *P*=3.05E-04, Figure 4A and 4B).

Cells with different expression of EphA10s and EphA10 were subjected to Western Blots for the analysis of regulation in ECAD complex (Figure 4C). Overexpressing EphA10s weakens the total expression of β-catenin while down-regulating EphA10 results in a decrease of pECAD and total β-catenin. Moreover, anti-β-catenin IP was employed to investigate the decrease of total β-catenin in the scenario (Figure 4D). Overexpressing EphA10s and down-regulating EphA10 recruit more membrane-associate β-catenin and apparently decreases cytoplasmic β-catenin, in which the β-catenin ratio of (M/CP) increases to 5.6 fold in EphA10s^+^/EphA10^−^cells (Figure 4E). Meanwhile, changes in the expression of EphA10s and EphA10 did not have significant impact on cell proliferation and apoptosis (Figure 4F and 4G).

### Higher EphA10s and lower EphA10 reverse lymph-node metastasis

**Figure 5:**
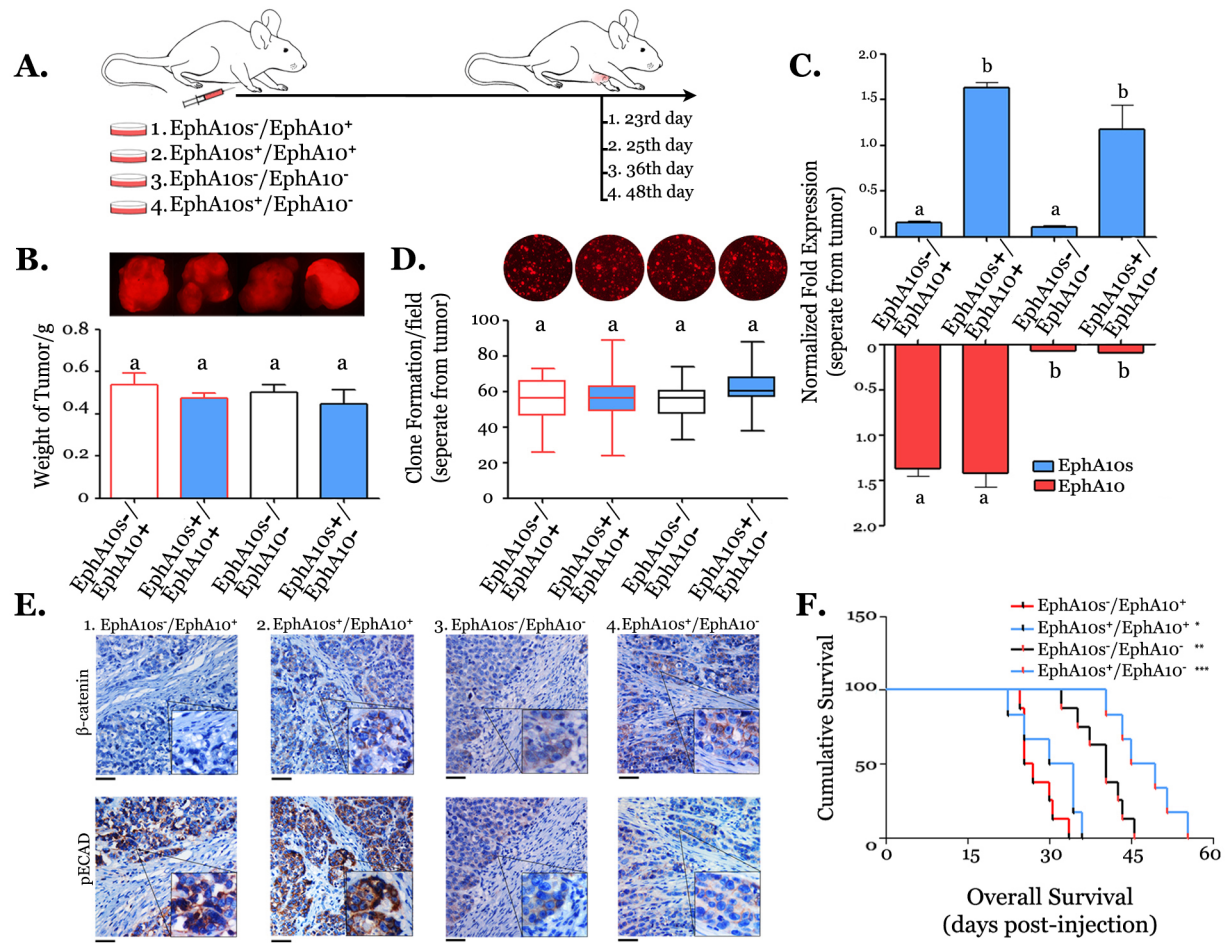
Xenograft model with tumors expressing different of EphA10s and EphA10 patterns **(A)** Recipients were injected with tumor cells with different EPHA10 isoforms expression patterns (mice:1. n=12; 2. n=10; 3. n=10; 4. n=10), and euthanized when the size of tumor mass grew to 8 mm × 8 mm. **(B)** Measurement of tumor mass in weight, overlapping tumor image of mCherry/phase are shown. **(C)** Measurement of mRNA of EphA10s and EphA10 in tumor mass. **(D)** Measurement of clone formation from a single cell, which was disassociated from tumor mass, sorted by mCherry label. Clones with mCherry image are shown above. Mean±SD is shown. **(E)** Consecutive slides of tumor mass were subjected to IHC staining with an antibody of pECAD and β-catenin. Representative images are shown with insert of 2×; scale bar: 200 μm. **(F)** Among each group (n=6), overall survival analysis were performed to evaluate the period from the day of tumor injection to the day of mice death. Statistic: the Log Rank test, two tails; * *P*=0.041 vs. EphA10s-/EphA10+, ** indicates *P*=0.026 vs. EphA10s+/EphA10+, and *** indicates *P*=0.01 vs. EphA10s-/EphA10-.

MDA-MB-231 cells with different EphA10s and EphA10 expression were employed into xenograft model (Figure 5A). Tumors with low EphA10s (EphA10s^−^) and high EphA10 (EphA10^+^) took a half of the time to grow until being sized as 8 mm × 8 mm than time that EphA10s^+^/EphA10^−^ tumors required (48 days, Supplementary Figure 5). Tumor mass with similar size was collected for weighting (Figure 5B), and re-identifying in expression pattern of EPHA10 isoforms (Figure 5C).

Tumors were trypsinized, then, single cancer cells were harvested by cell sorting. Different from the various growth rates *in vivo*, cells expressing different EphA10s and EphA10 show the similar capability to form clones *in vitro* (Figure 5D). Tumor slides were subjected to immunohistochemistry in pECAD and β-catenin (Figure 5E). Stronger membrane-associate staining of β-catenin was observed in higher EphA10s expressing tumors, while less accumulation of pECAD was found in lower Epha10 expressing tumors. Additionally, recipients with tumors were housed for overall survival analysis (Figure 5F). Mice with EphA10^−^ tumors show a significantly longer survival period, which is even better in the presence of additional EphA10s expression (*P*=0.01, Log Rank, HR=0.34 [95% CI: 0.065- 0.52], Figure 5F).

**Figure 6:**
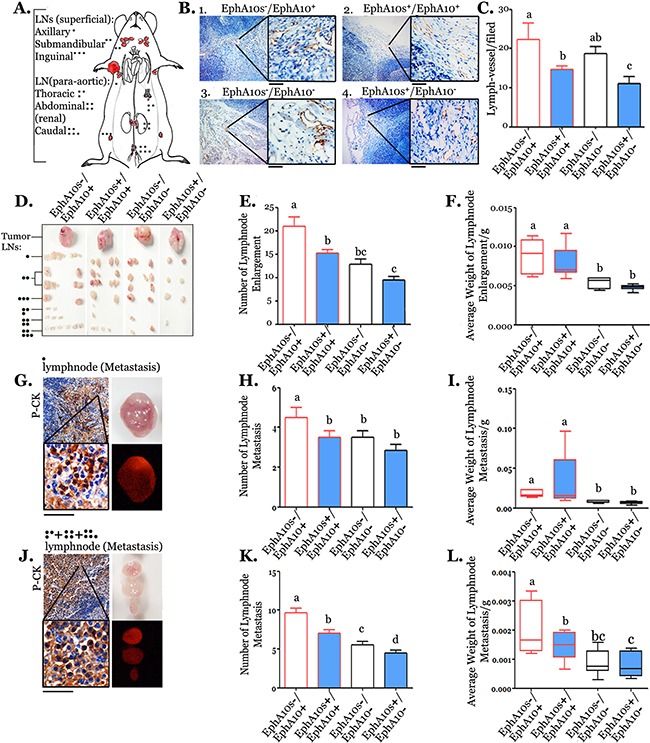
EphA10 and EphA10 expression pattern dominants lymph node metastases **(A)** A diagrammatic representation of the lymph metastases model is shown. **(B)** Peritumoral lymph vessels were detected by lymph vessel marker LYVE-1 in IHC. Insert is 5×; scale bar: 200 μm. **(C)** Measurement of number in detected peritumoral lymph vessels were performed. **(D)** Images of tumors and related enlarged lymph nodes from different section were shown. Measurement of number **(E)** and average weight **(F)** of total detected lymph nodes were performed. **(G)** Lymph node with metastasis from armpit was verified in IHC with an epithelial marker PCK staining, and shown in phase /mCherry fluorescent (expressing by tumor cells) representative image. Measurement of number **(H)** and average weight **(I)** in lymph nodes with metastasis from armpit were performed. In the same condition, lymph nodes from para-aorta were shown **(J-L)**. Insert is 5×; scale bar: 200 μm. Mean±SD is shown.

Measurement of lymph vascular invasion and lymphatic metastasis was performed when tumor had the similar size (Figure 6A). EphA10s^+^/EphA10^−^ tumors were found a 0.58-fold decrease in the formation of peritumoral lymph vessels than the ones in EphA10s^−^/EphA10^+^ tumors (Figure 6B and 6C). Visible (enlarged) lymph nodes from superficial or para-aortic area were measured in number and weight (Figure 6D-6F). In total, lymph nodes were found a decrease in terms of number (up to 0.53 fold) and average weight (up to 0.47 fold) from recipients with tumors expressing higher EphA10s and lower EphA10. Metastasis in enlarged lymph nodes, identified by staining with an epithelial marker pan-cytokeratin (PCK), was subjected to statistical measurement in number and weight, respectively (Figure 6G-6I). In axillary lymph nodes (sentinel), a decrease of 0.17 fold in number and 0.21 fold in average weight was observed in compared with ones from EphA10^+^ tumors. In para-aortic lymph nodes, tumor metastases show to be shrunken to 0.63 fold in number and 0.58 fold in average weight than the ones with EphA10s^−^/EphA10^+^ metastases. More details are presented in Supplementary Figure 6.

### Expression pattern of EphA10s and Epha10 linearizes the progression of breast cancer

**Figure 7:**
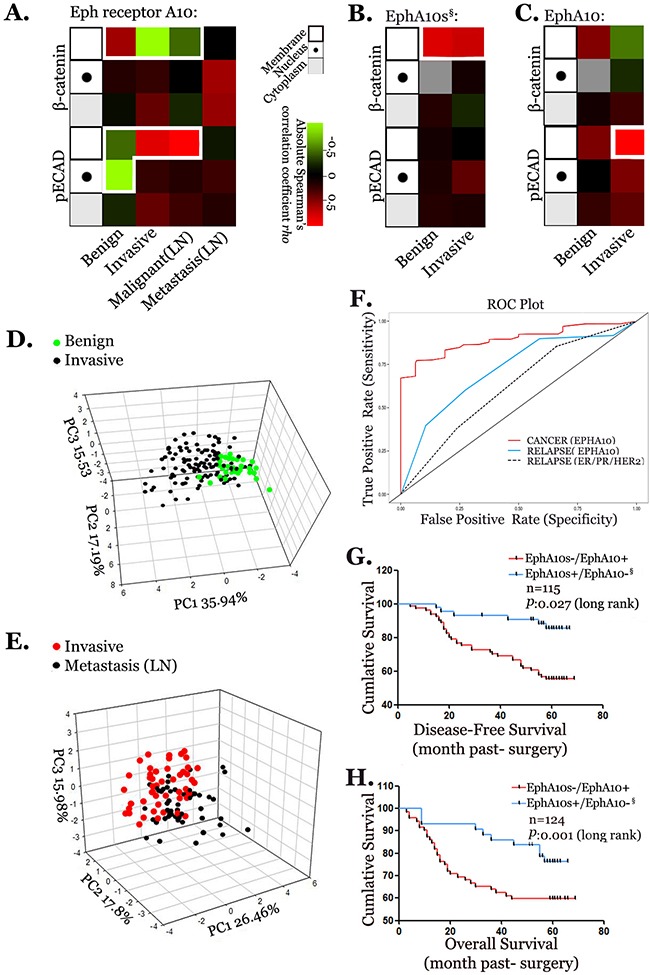
Profile of expression pattern of EPHA10 isoforms in the progression of breast cancer Co-expression analyses of Eph receptor A10 staining **(A)**, EphA10s **(B)**, EphA10 **(C)** with IHC staining of pECAD and β-catenin in membrane (frame), nuclear (dot), and cytoplasm (shade) among benign samples, invasive samples, invasive samples detected with lymph node metastasis (Malignant (LN)), and lymph nodes samples with metastasis (Metastasis (LN)). Statistics: SCCs with *P*≤0.05, values are colored (−1:green to 1: red). PCA was performed with EPHA10 profile in benign and invasive samples **(D)** and in invasive and metastasis samples **(E)**. Three-dimensional PCA score plot was shown. **(F)** ROC curve plots were performed to evaluate the Lasso prediction regression analysis with EPHA10 profile in cancer prediction (red), EPHA10 profile in cancer relapse prediction (blue), and ER/PR/HER profile in cancer relapse prediction (dashed). Disease free survival analysis **(G)** and overall survival analysis **(H)** were performed to analysis of the survival period between patients with high EphA10s/low EphA10 expression and patient low EphA10s/high EphA10 expression in a 5-year follow-up. § Samples were identified with high ephrin A5 expression.

Our findings *in vitro* suggested the interaction of EPHA10 isoforms with the complex of ECAD. Having the expression data of pECAD and β-catenin in 76 benign samples, 325 invasive samples, and 50 metastasis samples, which were matched with Eph receptor A10 staining and the expression of EphA10s and EphA10 in qualified 42 benign samples and 245 invasive samples, respectively, we generated Spearman's correlation coefficients (SSCs) analysis to illuminate the co-expression pattern, and annotated in subcellular distribution (Figure 7A-7C). In invasive samples, IHC staining shows a negative SCC with membrane-associate β-catenin and a positive value with membrane-associate pECAD. EphA10s positively associates with β-catenin in membrane in both benign and invasive samples, while EphA10 shows a positive value with membrane-associate staining of pECAD in invasive samples. The co-expression pattern suggested the interaction of EPHA10 isoforms with the complex of ECAD involved into BC progression.

Furthermore, we compiled the EPHA10 isoform profile, and employed principle component analysis (PCA) in the scenario of benign, invasive and metastatic samples (Figure 7D and 7E). The percentage of each PC lesion indicates its representative performance in diverse regions of a 3-dimensional version. The plots show that the profile of EPHA10 isoform properly describes and identifies the variousness between either benign and invasive samples, or invasive and metastatic samples.

The same profile and related expression profile of ECAD complex were used in Lasso regression analysis for a statistically potential of its prediction capability in the outcomes in 5-year follow-up (Supplementary Figure 7). The component is better promised when it is isolated earlier and/or valued with a larger number. In this model, Eph receptor A10 staining and EPHA10 isoforms show the strongest potential. Then, overall receiver operator characteristic (ROC) curves were employed to evaluate EPHA10-Lasso models (Figure 7F). In EPHA10 profile, ROC suggests a strong prognostic value of area under the curve (AUC) as 0.89 (95% CI: 0.86-0.93) in breast cancer prediction (red). When compared to the model built on the profile of ER, PR and HER2 signatures (dashed), our profile (blue) shows a better predictive efficiency as an AUC value of 0.72 (95% CI: 0.81-0.62) in tumor relapse (Figure 7F).

Furthermore, classical survival analysis was performed in the context of EphA10s and EphA10 expression (Figure 7G and 7H). Patients with higher EphA10s and lower EphA10 expressing demonstrate an advantage in analysis of non-disease survival (*P*=0.027, Log Rank, HR=0.26 [95% CI: 0.18-0.65]), and OS (*P=*0.001, Log Rank, HR=0.32 [95% CI: 0.19-0.66]) than ones with lower EphA10s and higher EphA10.

## DISCUSSION

We annotated the expression of EPHA10 protein with its isoforms as down-regulated EphA10s and high-expressed EphA10 in BC progression, where the stability of the complex of ECAD and β-catenin is disrupted.

Similar to Eph receptor family member [31], EPHA10 isoforms are suggested to participate in the cross talk between cells and the microenvironment through Eph/ephrin interaction and tyrosine kinase activity (Supplementary Figure 8). Secretary EphA10s stabilizes membrane-associated β-catenin and weakens cytoplasmic β-catenin (benign cell). Down-regulation of EphA10s results in weakening of β-catenin recruitment in membrane (cell), and progressing of cancer behavior (invasive cell). Eph receptor A10 domain is suggested to bind with ephrin A5 in this context. Activated ephrin A5 is functionalized to recruit membrane-associated β-catenin in epithelial cells [32, 33]. Our data suggested that overexpression of EphA10s lose to redistribute β-catenin when lack of ephrin A5. Whether the interaction of Eph receptor A10 domain and ephrinA5 requires another component as EphA2 is in research. EphA10 is redistributed in cytoplasm where it promotes tumor invasion and metastasis (invasive cell). EphA10 has a pseudo-RTK domain that is in low phosphorylation activity when binding with ECAD and pECAD. It is assumed to be a silencer to protect substrate from being phosphorylation by blocking the signal transduction. Membrane-associate EphA10 binds with ECAD (benign cell) while cytoplasmic EphA10 binds with pECAD (invasive cell). Down-regulated EphA10 results in the loss of protection, and a decrease of ECAD from membrane (benign cell), while a decline of cytoplasmic pECAD (invasive cell).

The diagnoses and therapies of BC are still in challenges [34]. Here, we highlighted the need of identification of EphA10s and EphA10 status. By envisaging a scenario whereby the expression patterns of EphA10s and EphA10 were altered in patients, their outcome was found variously. Tracing EPHA10 isoforms result in a potentially satisfied prediction of tumor development and progression. We reported the participation of EphA10s in lymph-vessel formation [35], and an anti-tumor role in the utility of endogenous or extraneous EphA10s. Moreover, cytoplasmic EphA10 played a strong role in promoting invasion and metastasis of cancer. A reduction of EphA10 *in vivo* significantly weakened tumor dynamic, and leads to a promising outcome. The signatures of EphA10s and EphA10 are, therefore, promising contributions to the modern diagnosis and drug development.

## MATERIALS AND METHODS

### Patients and TMAs

A total of 76 mammary gland cystadenoma samples and 275 invasive ductal carcinoma samples originating from patients who were treated at Tongji Hospital in Wuhan, China, from November 2008 to August 2009, were retrospectively enrolled, tumor-tissue microarrays (TMA: BR10010a) were purchased from Alenabio.com (Supplementary Table 4). Sections (5 μm) were cut from specimens with new scalpel for RNA extraction. A complete follow-up was available for 220 patients, ranging from 5 to 69 months (median 53 months, mean 60 months). This study was approved by the Ethics Committee of Tongji Hospital of Tongji Medical College, Huazhong University of Science and Technology, PR China.

### Cells and lentiviral transfection

MCF-10A and MDA-MB-231 cell lines were purchased from the American Type Culture Collection (USA) and cultured according to their instructions. All of cell lines were authenticated at the Shanghai Paternity Genetic Testing Center in April, 2013 using short tandem repeat (STR) DNA profiling (ABI 3130xl Genetic Analyzer, Life Technologies, USA). The plasmid (OHu08987) containing the EphA10s coding sequence (RefSeq Accession: NM_173641) was purchased from Genscript (USA). Lentivirus with mCherry label and carrying EphA10s plasmid or small hairpin RNAs of EphA10 were constructed and provided by CHENCHEM (China). Cell Infection was according to the manufacturer's instructions. Infected cells with mCherry signal were sorted using flow cytometer (Becton Dickinson, San Jose, CA).

### Migration and invasion assay

Assays were performed in Transwell chambers (Corning, NY) for 24 hours. Transwell filters were pre-coated with Matrigel (BD Biosciences) in the invasion assay. A total of 1-2×10^4^ cells were seeded in standard condition. Cells passing the filter were fixed and dyed with 0.1% crystal violet. The circle invasive assay (CIA) was performed similarly to invasion assay.

### RNA extraction and qPCR

Using a Tissue RNA FFPE Purification Kit (Promega, Madison, WI), total RNA of selected samples was extracted from qualified sections according to the manufacturer's protocol. RNA was purified on a Maxwell^®^ 16 instrument using a Total RNA Purification Kit (Promega, Madison, WI). An internal reference of GAPDH was using as previously described [36] (all primer sequences are in Supplementary Table 5).

### Western blot and co-IP

Standard Western Blot analysis was performed as previously described [37]. Three independent experiments in a certain condition were subjected to Western Blot analysis. Supernatant protein was extracted using an Amicon Ultra Centrifugal filter (Millipore). Membrane protein was extracted using a Mem-PER™ Plus Membrane Protein Extraction Kit (Life Technologies). Immunoprecipitation (IP) was performed using the Pierce Crosslink IP Kit (Thermo, USA) according to the manufacturer's protocol.

### Orthotropic human tumor xenografts

Female BALB/c nude mice (4 weeks old) were prepared based on previous studies [38, 39]. 2×10^5^ cells of MDA-MB-231 cells modified with different EphA10s and EphA10 expression patterns were injected under the 2nd breast fat pads. Mice were euthanized when the primary tumor grew to 8 mm×8 mm. Tumor masses and all visible lymph nodes were collected for further analyses.

### Data analysis

The SPSS statistical software package was used for all statistical analyses. For each parameter, SCCs were assessed to determine the co-expression among parameters. Disease-free and over-all survival was expressed as the number of months from surgery/injection to the occurrence of distant relapse or breast-related death by using Kaplan–Meier method and Log Rank test. In grouped comparisons, ANOVA was used followed by the least significant difference test for each group, and groups with different lowercase letters indicate significant differences. A P value of <0.05 was considered statistically significant. Mathematical models of PCA and Lasso linear regression [40] were performed in R.

Extended Materials and Methods with the associated references are in the Supplemental Material.

## SUPPLEMENTARY MATERIALS AND METHODS FIGURES AND TABLES


