# Supplementary material for: Isoform expression patterns of EPHA10 protein mediate breast cancer progression by regulating the E-Cadherin and β-catenin complex
Source: Oncotarget. 2017 Mar 6;8(18):30344–56. doi: 10.18632/oncotarget.15910 (PMC5444747; doi:10.18632/oncotarget.15910)
Supplement: Supplementary file 1 [file oncotarget-08-30344-s001.pdf]

Isoform expression patterns of EPHA10 protein mediate breast cancer progression by regulating the E-Cadherin and β-catenin complex

SUPPLEMENTARY MATERIALS AND METHODS

Reagents

Antibodies included: Eph receptor A10 (Genetex, Irvine, CA), phospho-tyrosine (R&D systems, Minneapolis, USA), ECAD (Abcam, Cambridge), pECAD (Abcam, Cambridge), β-Catenin (Abcam, Cambridge), Phospho-β-Catenin (Abcam, Cambridge), ephrin A5 (EFNA5) (Genetex, Irvine, CA), p120ctn (Abcam, Cambridge), EGFR (Abcam, Cambridge) GAPDH (Santa Cruz, CA), LYVE-1 (Waltham, MA) and pan-cytokeratin (PCK) (Waltham, MA). The reagents CM-DIL, MG132, BFA, AG490 and Genistein were purchased from Beyotime (China).

Patients and TMAs

All IDC tissues are from patients primarily diagnosed as IDC and treated with surgery followed by chemotherapies. Tissues were obtained by experienced surgeons and were immediately frozen in liquid nitrogen. Diagnoses of IDC were established by two independent pathologists.

Animals

Female BALB/c nude mice (4 weeks old) were purchased from Beijing HFK Bio-Technology Co, Ltd. for studies approved by the Committee on the Ethics of Animal Experiments of Tongji Medical College. The mice were maintained in the accredited animal facility of Tongji Medical College.

Cells culturing and drug treatment

Cell lines of MCF-10A, MDA-MB-231, MCF-7, Hcc1937, MDA-MB-436, MDA-MB-435s, SKBR3, and T47D were purchased from the American Type Culture Collection (USA). MCF-7 cells were maintained in DMEM medium (Gibco) supplemented with 10 µg/ml insulin (bovine); MDA-MB-436 cells were cultured in L-15 medium (Gibco) supplemented with 10 µg/ml insulin (bovine); MDA-MB-435s cells were cultured in L-15 Medium (Gibco); Hcc1937 cells were cultured in RPMI-1640 Medium (Gibco); SKBR3 cells were maintained in McCoy's 5A modified medium (Invitrogen, Eugene, OR, USA)); and T47D cells were maintain in RPMI-1640 Medium, respectively. All medium were added within 10% fetal bovine serum and antibiotics for cell culturing. And all cells used were grown at 37 °C with 5% CO2. When using drug of AG490 (20µM) [1] and Genistein (50µM) [2], cell were incubated with media in 6 hours, respectively.

IHC scoring

Sections (5 µm) from paraffin-embedded tissues of patients, TMAs, recipient mice were subjected to staining with Eph receptor A10 (Genetex, Irvine, CA), ECAD-p (abcam, Cambridge, UK), and β-catenin (abcam, Cambridge, UK), as primary antibodies, which were used in 1:500, 1:1000, 1:1000 dilution in IHC, respectively. For imunocytochemistry, cells were fixed by using 4% paraformaldehyde for 15 min, and applied with primary antibodies accordingly. Evaluation of staining was performed by two pathologists blinded both to the clinical and molecular data. Both cytoplasm and nuclear staining of Eph receptor A10, ECAD-p, and BCTN were described in terms of intensity (0: negative; 1: weak; 2:intermediate; 3: strong) and distribution (1:<1%; 2:1%-10%; 3: 10%–33.3%; 4:33.3%-66.6%; 5: >66.6%) according to the Allred's score [3]. Total score was given by combining with both intensity value and its distribution, considering an A-Score of 0 or 2-8. An Allred score of >6 was considered as positive (overexpression). Membrane staining of BCTN and ECAD-p was scored according to a previously used membrane scoring system in ECAD [4]. Briefly, the proportion of staining cell with complete membrane's staining was grouped into 4 categories: 0:0-10%; 1: 10%–25%; 2: 25%–50%; 3: 50%–75%; and 4: >75%.

Cell viability, apoptosis assays and colony forming assays

Cells were labeled with 5mM Carboxyfluorescein diacetate succinimidyl ester (CFSE) (CellTraceTM CFSE Cell Proliferation Kit, Invitrogen, Carlsbad, CA, USA) for 10 min, at 37°C in the dark, following by washing for three times in PBS. CFSE labeled cells were seeded and keep incubating in darkness for 3 days. Intensity of fluorescein of cultured cell was quantified by BD FACSCalibur (BD Biosciences, San Jose, CA, USA) at channel FL1-H with compared to their primary intensity. The geometric mean of its fluorescence intensity was calculated as the degree of cell proliferation. Cells were stained with Annexin V and propidium iodide (PI) and the percentage of apoptotic

cells was determined by flow cytometry. All results of cytometry were analyzed by using FlowJo software. 1×10^3 cells sorted cells were subjected to colony forming assay for 7 days with regular medium.

Sample screening for RNA extraction

Mounted 5μm serial sections were developed for each sample and HE staining was performed. Base on HE staining, sections with>25% mammary gland cells and >75% of purity of neoplastic cells were chose for RNA extraction and await analyses. Out of 42 mammary gland tissues and 245 IDC tissues were selected. Selected samples were extracted from sections according to the manufactory's protocol of Tissue RNA FFPE Purification Kit (Promega). Briefly, tissue sections were scraped into 1.5 ml microcentrifuge tubes, respectively. Mineral Oil was added into each sample tube and was heated in 80°C, 2 minutes for a better mixture of RNA and other materials. Lysis Buffer, Proteinase K and Blue Dye were mixed and added into each tube to digest protein included in tissues and heated in 80°C, 1 hour. Following by DNase treatment at room temperature for 15 minutes, aqueous phase of each tube was transferred into prepared cartridge.

REFERENCE

1. Yan-xia Zhang, Li Yan, Guang-yu Liu, Wen-jun Chen, Wei-hong Gong, and Jin-ming. Inhibition of janus kinase 2 by compound AG490 suppresses the proliferation of MDA-MB-231 cells via up-regulating SARI. Iran J Basic Med Sci. 2015;18: 599–603.
2. Su Y, Simmen RC. Soy isoflavone genistein upregulates epithelial adhesion molecule E-cadherin expression and attenuates beta-catenin signaling in mammary epithelial cells. Carcinogenesis.2009; 30:331-339.
3. Allred DC, Harvey JM, Berardo M, Clark GM. Prognostic and predictive factors in breast cancer by immunohistochemical analysis. Mod Pathol.1998; 11:155-168.
4. Mahler-Araujo B, Savage K, Parry S, Reis-Filho JS. Reduction of E-cadherin expression is associated with non-lobular breast carcinomas of basal-like and triple negative phenotype. J Clin Pathol.2008; 61:615-620.

SUPPLEMENTARY FIGURES AND TABLES

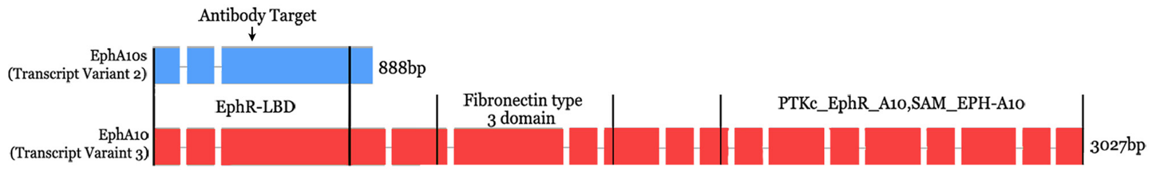

Supplementary Figure 1: The coding sequence (CDS) and alignments of EphA10s (bule) and EphA10 (red) were noted with organizational protein function domains (Gene Database) indicated.

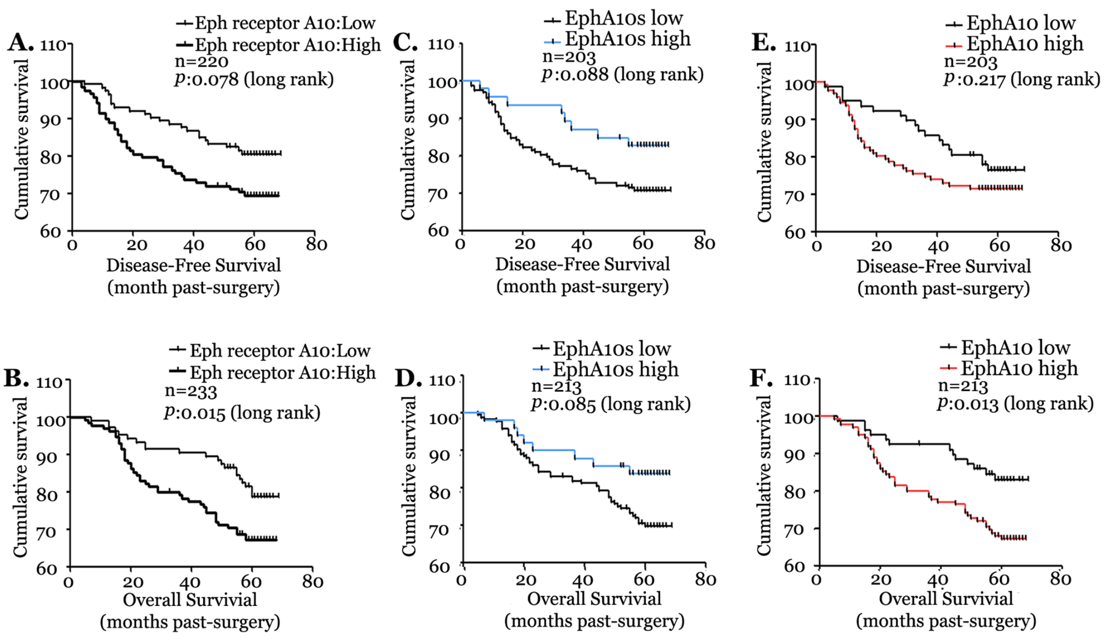

Supplementary Figure 2: Survival analysis determined by EPHA10 protein. Disease-free and overall survival and survival analysis of invasive samples according to Eph receptor A10 staining **(A)** and **(B)**; EphA10s expressing **(C)** and **(D)**; EphA10 expressing **(E)** and **(F)**. Statistics: Kaplan-Meier in Log Rank, two-tailed.

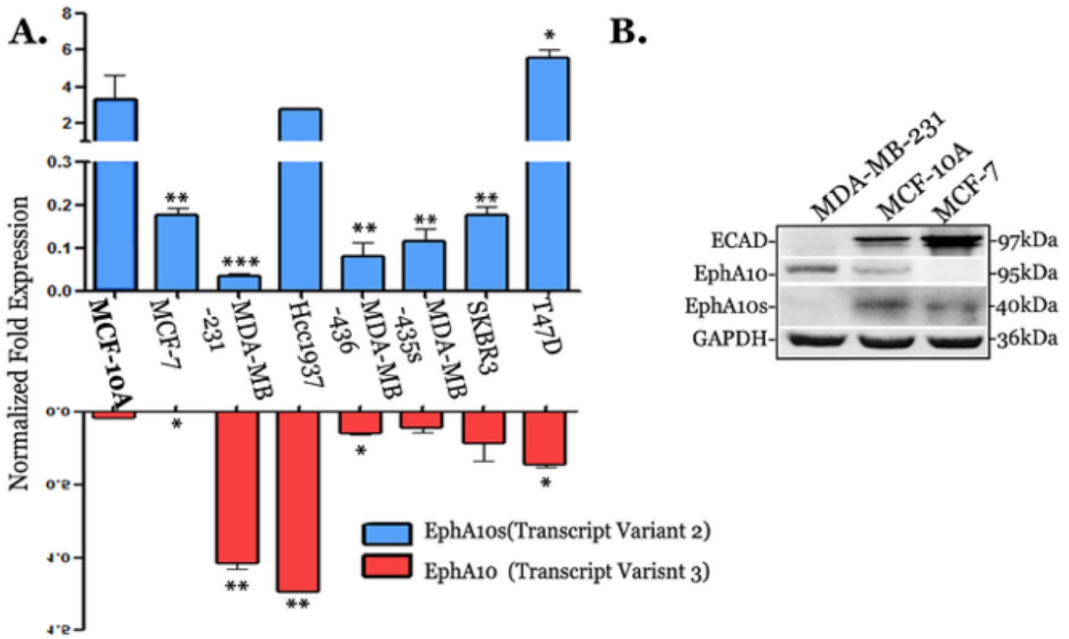

Supplementary Figure 3: EPHA10 protein expression in breast cancer cell lines. **(A)** mRNA expression of EphA10s (blue) and EphA10 (red) in breast cancer cell lines. Statistics: one-way ANOVA, two-tailed; vs. expression in MCF-10A, * $P<0.05$, ** $P<0.01$, *** $P<0.001$; Mean±SD is shown. **(B)** MDA-MB-231 cells (invasive), MCF-10A cells (benign), and MCF-7 cells (less invasie) were subjected to analysis of ECAD, EphA10 and EphA10s in Western Blot. GAPDH is used as loading control.

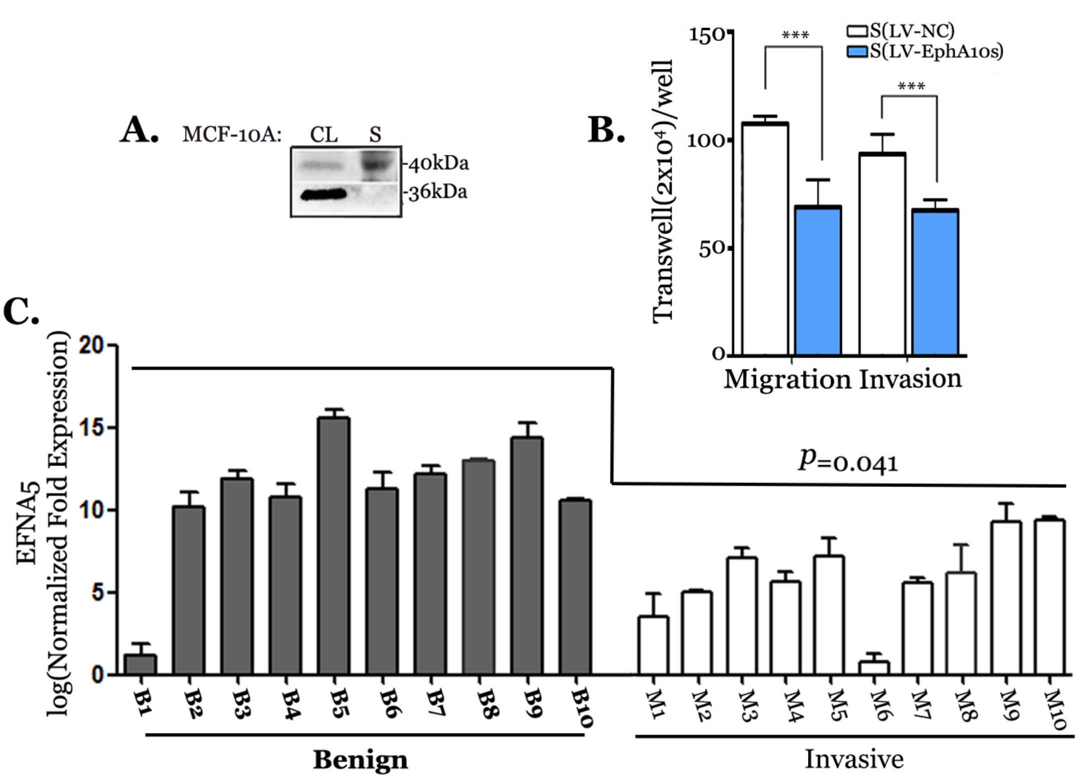

D.

EFN A5(qPCR) Corralation	Benign		Invasive	
	r	*p* value	r	*p* value
qPCR:EphA10s	0.169	0.296	0.558	**0.004**

Supplementary Figure 4: EphA10s expression and clinical correlation with EFNA5. (A) MCF-10A cell lysates (CL) and cell supernatant (S) were subjected to analysis of EphA10s expression in Western Blot. GAPDH is used as control. **(B)** Measurement of migration and invasion was performed in cells cultured with medium from MDA-MB-231 cells and EphA10s expression MDA-MB-231 cells (blue) in transwell chamber for 24 hours, 3 independent repeats, ****P*<0.001 **(C)** mRNA expression of ephrin A5 in 10 random benign or invasive samples. Statistics: one-way ANOVA, two-tailed; Mean±SD is shown. **(B)** Spearman's correlation coefficient between mRNA expression of EphA10s and ephrin A5 in benign and invasive samples.

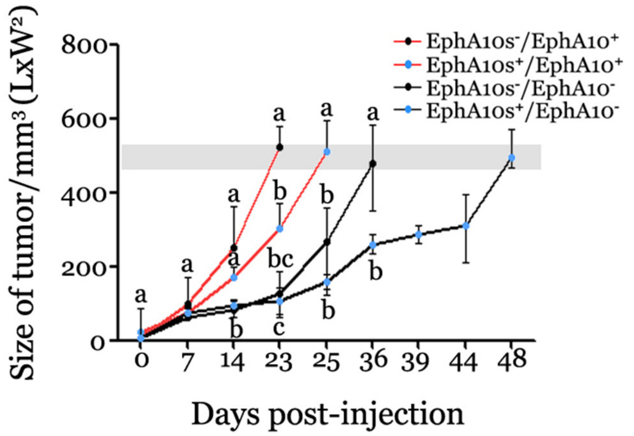

Supplementary Figure 5: The growth of tumor mass expressing different EPHA10 isoform patterns in xenograft model.

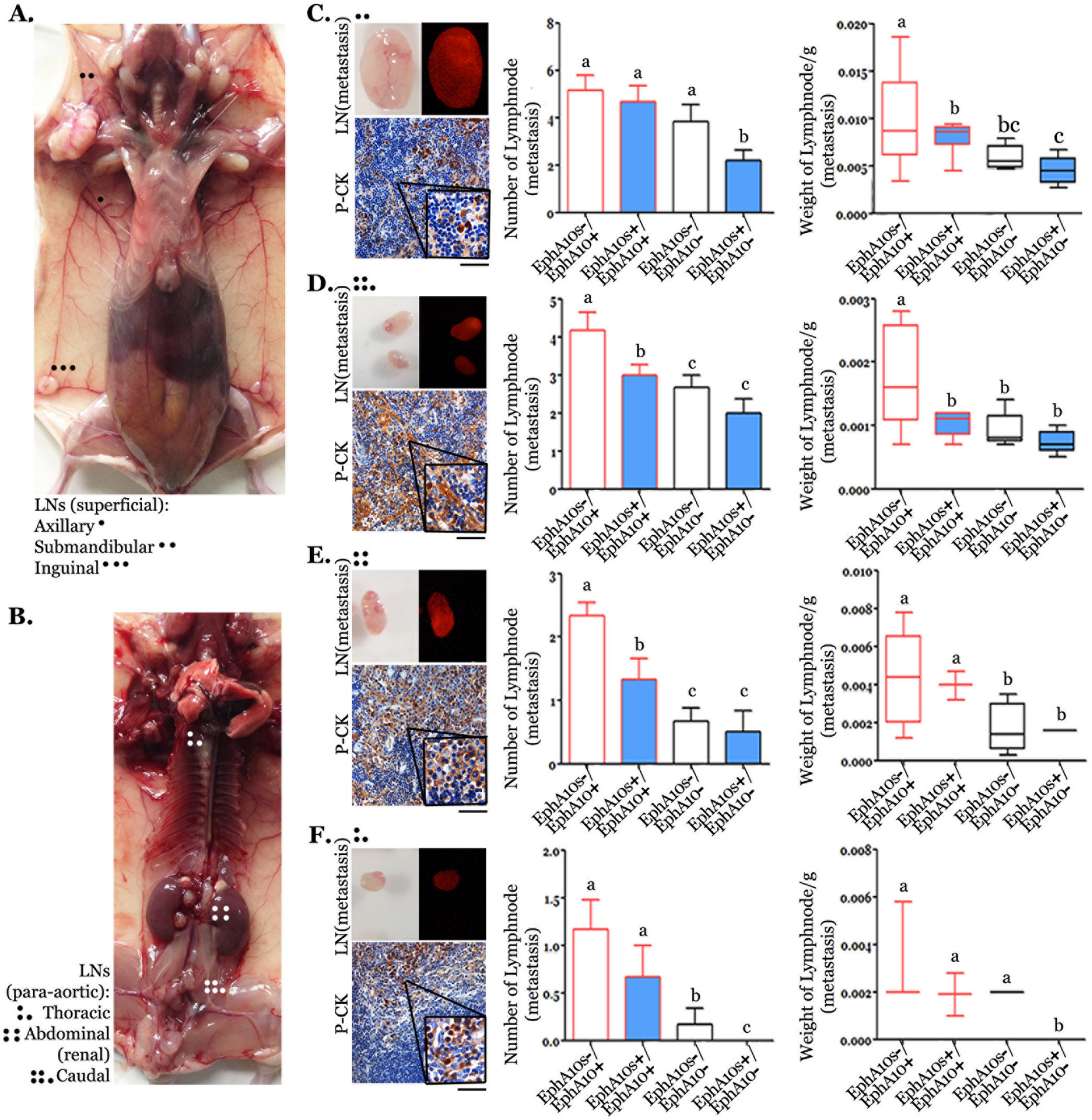

Supplementary Figure 6: Lymph node metastasis of tumor with different expression pattern of EPHA10 isoform. Anatomy image is shown and the section where lymph node were collected is noted with different blot patterns, **(A)** superficial zone (axillary, submandibular, inguinal) and **(B)** para-aorta (thoracic, abdominal, and caudal). Visible lymph node was subjected to immunohistochemistry staining with an epithelial marker PCK. Inset is 5 ×; scale bar: 20 µm. Image of phase/mCherry in lymph node with metastasis is shown. The measurement of number and average weight were performed in lymph nodes with metastasis, Mean±SD is shown. **(C)** submandibular, **(D)** caudal; **(E)** renal; **(F)** thoracic.

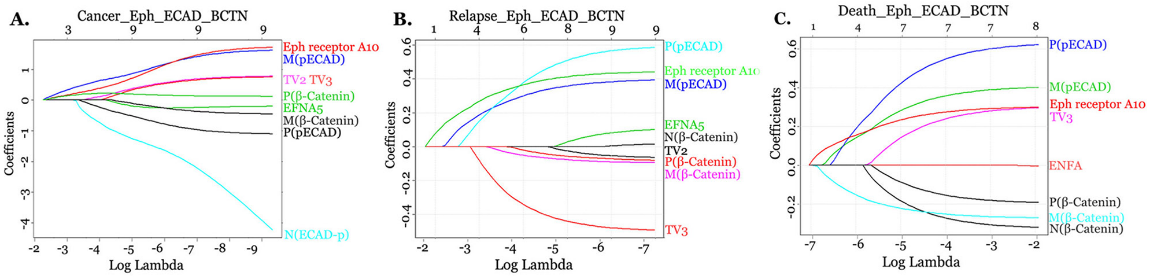

Supplementary Figure 7: Lasso prediction regression model with profile of EPHA10 expression in breast cancer, (A) cancer, (B) relapse, (C) death in 5-year follow-up.

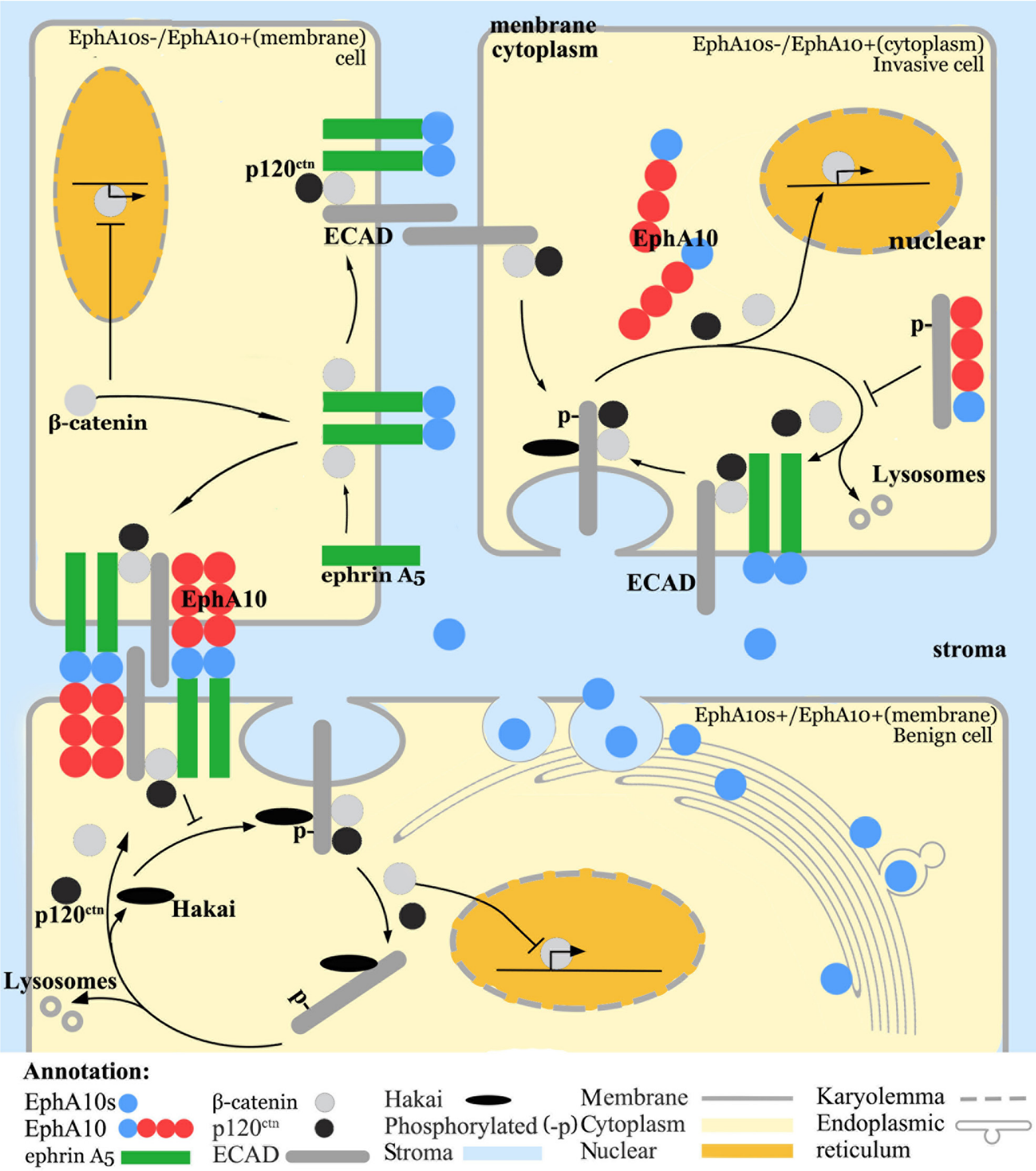

Supplementary Figure 8: Summary of the malignant transformation with expression pattern change of EPHA10 protein. Benign cell (bottom and up left), invasive cell (up right) are envisaged. Benign cell (bottom) with high EphA10s expression and membrane-associate EphA10 expression maintains the stability of the complex of ECAD and β-Catenin. Cell (up left), while low EphA10s and membrane-associate EphA10 performs a request of EphA10s in need of β-Catenin recruitment. Invasive cell (up right) with low EphA10s and cytoplasmic EphA10 shows a loss of cell-cell connection, disruption of the complex of ECAD and β-Catenin, activation of oncogene expression due to the loss of protection from EphA10s and membrane-EphA10.

Supplementary Table 1: Profile of Eph receptor A10 (IHC) in the development and progression of breast cancer

Traits		IHC:EPHA10		P value
		High	low	
	n	n(%)	n(%)	
Benign	76	4(5.36)	72(94.7)	**0.000**
Invasive	275	167(60.7)	108(39.3)	
Total	351			
Invasive	50	36(72.0)	14(28.0)	**0.017**
Metastasis(LN)	50	46(92.0)	4(8.00)	
Total	100			

P value is evaluated with Pearson Chi-square, two tails.

Supplementary Table 2: Profile of Eph receptor A10 (IHC) in breast cancer characters

Traits		IHC:EPHA10		P value	Traits		IHC:EPHA10		P value
		High	low				High	low	
	n	n(%)	n(%)			n	n(%)	n(%)	
Invasive	275				Metastasis(LN)	50			
**AGE**	260				**AGE**	50			
≥45	182	123(67.6)	59(32.4)	0.157	≥45	37	27(73.0)	10(27.0)	1.000
<45	78	45(57.7)	33(42.3)		<45	13	9(69.2)	4(20.8)	
**pT**				268	**pT**	50			
T1 and T2	218	126(57.8)	92(42.2)	**0.024**	T1 and T2	37	25(67.6)	12(32.4)	0.303
T3 and T4	50	38(76.0)	12(24.0)		T3 and T4	13	11(84.6)	2(15.4)	
**pN**	268				**pN**	50			
N0	207	125(60.4)	82(39.6)	0.656	N1	35	22(62.9)	13(37.1)	**0.039**
N1 and N2	61	39(63.9)	22(36.1)		N2	15	14(93.3)	1(6.70)	
**pSTAGE**	268				**pSTAGE**	50			
I and II	222	132(59.5)	90(40.5)	0.245	II	34	21(61.8)	13(38.2)	**0.021**
III	46	32(69.6)	14(30.4)		III	16	15(93.8)	1(6.25)	
**Molecular Typing**	253				**Molecular Typing**	47			
Luminal A and B	152	91(55.9)	61(44.1)	0.762	Luminal A and B	18	13(72.2)	5(27.8)	0.598
HER-2	85	55(64.7)	30(35.3)		HER-2	16	11(68.8)	5(31.2)	
Basal-like	16	10(62.5)	6(37.5)		Basal-like	13	11(84.6)	2(15.4)	

Indications of pathological parameters are as follow:
Tumor-node-metastasis (TNM) is classified according to the revised American Joint Committee on Cancer TNM classification.
P value is evaluated with Pearson Chi-square, two tails

Supplementary Table 3: mRNA expression of EphA10s and EphA10 in benign and invasive samples

Traits		qPCR:EphA10s		P value		qPCR:EphA10		P value
		High	Low			High	Low	
	n	n(%)	n(%)		n	n(%)	n(%)	
Benign	42	18(42.9)	24(57.1)	**0.000**	42	14(33.3)	28(66.7)	**0.000**
Invasive	245	31(12.7)	214(85.4)		245	173(70.6)	72(29.4)	
Total	287				287			

P value is evaluated with Pearson Chi-square, two tails.

Supplementary Table 4: Profile of ER, HER2 and PR in invasive and metastasis samples

	Invasive Cases (%)	Metastasis Cases (%)	Total (%)
**ER**	144 (56.5)	14 (28.0)	158 (51.8)
**PR**	140 (54.9)	16 (32.0)	156 (51.1)
**HER-2**	204 (80.0)	24 (48.0)	228 (74.8)
**Total**	488	54	542

Supplementary Table 5: Primer sequences

Primer name	Forward	Reverse
**GAPDH**	GACAGTCAGCCGCATCTTCT	TTAAAAGCAGCCCTGGTGAC
**TV 2**	GAGAGAGGGATTGGGAAGTTG	CCACAGGCAAGAGAGAGAATG
**TV 3**	CCCAGGGTTTTACAAGGTGTC	GAGCGGCTCAGGCTGTACT
**EFN A5**	ACATGGTGAACTTTGATGGCTACA	CGGTTACATTCCCATCTCTTGAA
**BCTN**	GAGCCTGCCATCTGTGCTCT	ACGCAAAGGTGCATGATTTG
